# Supplementary material for: Genome-wide identification of 5-methylcytosine sites in bacterial genomes by high-throughput sequencing of MspJI restriction fragments
Source: PLoS One. 2021 May 11;16(5):e0247541. doi: 10.1371/journal.pone.0247541 (PMC8112702; doi:10.1371/journal.pone.0247541)
Supplement: S1 File — (PDF) [file pone.0247541.s001.pdf]

S1 Text. Description of S1 through S4 Data Sets.

These data sets comprise FASTA files from which the motifs in Tables 5 and 6 were determined. A representative set is included, and any files not included here are available upon request from the authors. Trimmed and merged reads from Illumina or Ion Torrent were processed in the following manner: duplicate reads were merged into a single entry, and all reads were mapped to (1) a reference sequence, (2) the MFRE activator oligonucleotide, and (3) sequences of size standards used in the gel purification process; only exact matches were marked as such. The results of this process were written to the "processed" FASTA files included here.

FASTA identifiers have the following format: "> source file | match information | read length | number of copies in the original read set." Those reads where the match information begins with the word "reference", all grouped at the start of the file, are exact reference matches and were used for motif identification. All others (labeled as "activator", "marker_plasmid", "no matches", etc.) were ignored. Reference sequences used for matching are as described in Table S5 with the exception of the M.AvaII clone, for which *E. coli* BL21(DE3) was used.

File names are in the following format: "genome_job-number_cleavage-enzyme_processed.fasta". The relevant portions are the genome (as given in Tables 5, 6, and S5) and the cleavage enzyme ("MspJI", "FspEI", "MF" for MspJI+FspEI in combination, or "MFL" for MspJI+FspEI+LpnPI in combination).

Contents of the data set files are as follows:

<u>S1 Data Set (13 files):</u>
A. calcoaceticus (MspJI)
A. hydrophila (FspEI)
A. flos-aquae (MspJI, FspEI)
Arthrobacter sp. NEB688 (MspJI)
Bacillus sp. N3536 (MspJI)
B. marisrubri (MFL)
Clone M.AvaII (MspJI)
D. radiodurans (MspJI, FspEI)
R. sphaeroides 2.4.1 (MF)
R. sphaeroides CH10 (MF)
X. badrii (MspJI)

<u>S2 Data Set (3 files):</u>
A. gelatinovorum (MspJI, FspEI)
A. citreus (MF)

<u>S3 Data Set (5 files):</u>
B. megaterium (MspJI)
B. stearothermophilus (MspJI)
Moraxella sp. ATCC49670 (MF)

S. denitrificans (MspJI, FspEI)

S4 Data Set (3 files):
E. coli DHB4 (FspEI)
S. cremoris (MspJ, FspEI)

S2 Text. The use of sequence logos for visualization of MFRE-Seq data.

S7 Table shows sequence logos corresponding to all of the motifs shown in Table 5. Each logo was derived from all putative CCMD reads of the (16,16) length corresponding to the motif in question. The read length and number of reads for each used are shown in the table.

Sequence logos have the advantage of visualizing certain sequence features not readily discernable from the computationally determined motif or nucleotide distributions. For example, an SSN repeat context is apparent around the methylated CTCGAG and TGCA sites of *Halorubrum*. In addition, the sequence requirements of the MspJI cleavage enzyme are easily visualized in the case of the GATC motif from *A. calcoaceticus*, the GCGC motif of the M.HhaI clone, and the GGWCC motif of the M.AvaII clone. Finally, the lack of sequence context in the case of FspEI-cleaved *A. variabilis* genomic DNA shows why the CGATCG motif was not determined automatically.

However, the logos can also be misleading, as in the case of the RCCGGY motif of MspJI-cleaved *A. variabilis*, in part because it does not account for the dependencies between bases. Although the logo suggests a motif of RCHDGY, this is due to the presence of reads of other origin. S8 Table, which breaks down the representation of all possible RCHDGY sites in *A. variabilis*, shows that those sequences conforming to RCCGGY (in red) are almost completely methylated (as determined by MFRE-Seq reads), while all other sequences are not.

S1 Table. Base filtering of selected read structures containing CCWGG (l_{-2} = 31) or CCWGG (l_{-4} = 29).

Motif[a]	Read class	Read length	Base-Filter Pass
**C**CWG**G**	(16,16)	29	Yes
C**C**WG**G**	(16,17)	30	Yes
C**C**W**G**G	(16,16)	31	Yes
CC**WG**G	(16,17)	32	No
C**C**WG**G**	(15,16)	30	Yes

[a] Methylated bases are underlined, and bases used for base filtering are in boldface.

S2 Table. Number of (all / base-filtered) reads of various classes for lengths 30-32 bases.

Class	length 30	length 31	length 32
**(–13,18)**	48/0	–	–
**(–13,19+)**	74/1	297/6	114/16
**(14,17)**	1563/0	–	–
**(14,18)**	–	16/0	–
**(14,19+)**	–	–	12/0
**(15,17)**	–	19,919/0	–
**(15,18)**	–	–	335/0
**(15,16)**	120,429/120,429	–	–
**(16,16)**	–	4,161,371/4,161,371	–
**(16,17)**	–	–	1,440,996/0
**Total**	122,114/120,430	4,181,603/4,161,377	1,441,457/16

S3 Table. Comparison of enzyme activator oligonucleotides.[a]

		Exact Reference Matches		Exact Activator Matches	
**DNA**	**Activator[b]**	**Total**	**NR**	**Total**	**NR**
B.sp.	none	118,676	54,577	2	2
B.sp.	standard	167,275	70,275	1069	36
B.sp.	N	79,810	42,902	2	2
B.sp.	U	230,808	89,091	124	10
B.sp.	NU	159,540	69,711	3	2
P.men.	none	1,774	1647	8	6
P.men.	standard	169,887	141,390	1185	37
P.men.	N	154,787	130,047	3	2
P.men.	U	133,338	111,331	42	11
P.men.	NU	175,246	146,740	9	7

[a] Numbers of total and unique (“NR” = non-redundant) reads identified from Ion Torrent sequencing of libraries from digests of two genomic DNA samples using various activator oligonucleotides.
[b] Standard activator = CTGC$\underline{C}$AGGATCTTTTTTGATC$\underline{C}$TGGCAG;
activator N = (c6)-CTGC$\underline{C}$AGGATCTTTTTTGATC$\underline{C}$TGGCAG;
activator U = CTGC$\underline{C}$AGGATCUUUUUUGATC$\underline{C}$TGGCAG;
activator NU = (c6)-CTGC$\underline{C}$AGGATCUUUUUUGATC$\underline{C}$TGGCAG;
where $\underline{C}$ = m5C, U = deoxyuracil, and (c6) = 5'-amino modifier C6.

S4 Table. Motif calling by random downsampling of *E. coli* DHB4 reads of length 31 and copy number ≥ 2.

Downsample Factor	Total Input Reads (l_{-2}=31)	Reads Used (l_{-2}=31, cn≥2)	Motif
1	17,084	11,714	CCWGG
5	3,416	2,323	CCWGG
25	683	458	CCWGG
125	136	96	CCWGG
250	68	54	CCWGG
500	34	28	CCWGG
550	31	23	VNNNNNNNNCCWGG

S5 Table. Summary of m5C motifs identified in Tables 5 and 6.

Motif	No. Genomes	m5C Arrangement (x)	CCMD Length (l_x)
AGCT	1	+1	34
CCGC[a]	1	–2	31
CCGG	1	–3	30
CCGG	2	–1	32
CGCG	2	–3	30
GATC	4	+3	36
GCGC	1	–1	32
TGCA	1	+1	34
CCNGG	2	–2	31
CCWGG	1	–4	29
CCWGG	5	–2	31
GGNCC	2	+4	37
GGWCC	2	+2	35
CACGTG	1	–1	32
CGATCG	3	–5	28
CTCGAG	1	–1	32
GCCGGC	2	–1	32
GCRYGC	1	–3	30
GCTAGC	1	–3	30
GGNNCC	1	+3	36
RCCGGY	3	–3	30
YCGCGR	1	–3	30
RCCWGGY	1	–2	31
CRCCGGYC	1	–3	30

[a] Non-palindromic.

S6 Table. Motifs discovered or confirmed with MFRE-Seq and the enzymes responsible.

Organism	Motif	Enzyme	Previously Characterized[a]	GenBank Acc.
Acinetobacter calcoaceticus ATCC49823[b]	CGCG	M.AccII	R (Mm)	CP050993
Acinetobacter calcoaceticus ATCC49823[b]	GATC	*unassigned*	–	
Aeromonas hydrophila	GCCGGC	M.AhdIII	–	CP050994
Agrobacterium gelatinovorum[c]	ACCGGT	M.AgeI	R (Mm)	CP051181
Agrobacterium gelatinovorum[c]	CCWGG	*unassigned*	–	
Anabaena flos-aquae CCAP 1403/13f[d]	GGNCC	*unassigned*	–	CP051206
Anabaena flos-aquae CCAP 1403/13f[d]	RCCGGY	M.AflIX	–	
Anabaena variabilis ATCC27893[e]	CGATCG	M.AvaVIII	Mm	BA000019
Anabaena variabilis ATCC27893[e]	RCCGGY	M.AvaIX	Mms	
M.AvaII clone (*A. variabilis* ATCC27893)	GGWCC	M.AvaII	R (Mm)	BA000019
Arthrobacter citreus NEB577	CCGC	M.AciI	R (Mm)	CP053690
Arthrobacter sp. NEB688	AGCT	M.AscII	–	CP053707
Bacillus megaterium S2	GCTAGC	M.BmtI	R (Mm)	CP051128
Bacillus megaterium S2	GATC	M.BmtII	–	
Bacillus sp. N3536	GATC	M.BscXII	R (Mm)	CP046057
Bacillus stearothermophilus CPW16[f]	RCCGGY	M.BsrFI	R (Mm)	CP051164
Bifidobacterium kashiwanohense APCKJ1[g]	CCWGG	M.BkaJ1I	–	CP026729
Bermanella marisrubri	CCWGG	M.Bma65I	–	CP051183
Deinococcus radiodurans[h]	YCGCGR	M.DrdVII	–	CP031163
E. coli DHB4	CCWGG	M.EcoDHB4Dcm	Mms	CP014270

M.HhaI clone (*Haemophilus haemolyticus*)	GCGC	M.HhaI	Mms	CP038817
Halorubrum sp. BOL3-1<br>*Halorubrum* sp. BOL3-1	CTCGAG<br>TGCA	*unassigned*<br>*unassigned*	–<br>–	CP034692 and<br>CP034693
Moraxella sp. ATCC 49670[i]	CCGG	M.MspI	Mms	CP051211
Neisseria meningitidis 95/134<br>*Neisseria meningitidis* 95/134<br>*Neisseria meningitidis* 95/134	GGNNCC<br>GCRYGC<br>CCWGG	M.Nme95II<br>M.Nme95III<br>M.Nme95IV + M.Nme95V	–<br>–<br>–	CP021725
Pseudomonas maltophilia[j]<br>*Pseudomonas maltophilia*[j]	CACGTG<br>RCCWGGY	M.PmlI<br>*unassigned*	R (Mm)<br>–	CP051467
Pseudomonas mendocina	GGWCC	M.PmeII	R (Mm)	CP027657
Pseudomonas sp. OM2164[k]	CCWGG	M.PspOMVI	–	CP051542
Rhodobacter sphaeroides 2.4.1	CGATCG	M.Rsp241I	–	CP030272
Rhodobacter sphaeroides CH10	CGATCG	M.RspCH10I	–	CP051469
Streptococcus cremoris F[l]	CCNGG	M1.ScrFI + M2.ScrFI	Mm	CP051518
Sulfurimonas denitrificans DSM1251	CCNGG<br>GATC<br>CGCG<br>CCGG	M.SdeAII<br>M.SdeAVI<br>M.SdeAORF121P<br>M.SdeAORF1839P	Mm<br>Mm<br>Mm<br>Mm	CP000153
Xanthomonas badrii[m]	CRCCGGYG	M.XbaIV	–	CP051651

[a] "Mm" = MTase motif was known from previous work, but methylated base was not; "R (Mm)" = MTase motif could be inferred from that of a characterized cognate REase; "Mms" = MTase motif and specific methylated base were known from previous work and confirmed here; "–" = neither motif nor methylated base were known previously and were newly determined here.

[b] Now renamed to *Chryseobacterium* sp. NEB161.

[c] Now renamed to *Thalassobius gelatinovorus* NEB572.

[d] Now renamed to *Dolichospermum flos-aquae* CCAP 1403/13F.

[e] Now renamed to *Nostoc* sp. PCC 7120.

[f] Now renamed to *Geobacillus subterraneus*.

[g] Now renamed to *Bifidobacterium catenulatum* subsp. *kashiwanohense* APCKJ1.

[h] Now renamed to *Deinococcus wulumuqiensis* NEB479.

[i] Now renamed to *Acinetobacter* sp. NEB149.

[j] Now renamed to *Stenotrophomonas maltiphila* NEB515.

[k] Now renamed to *Paracoccus sanguinis* OM2164.

[l] Now renamed to *Lactococcus lactis* subsp. *cremoris* F.

[m] Now renamed to *Xa*

S7 Table. Sequence logos corresponding to the motifs identified in Table 5, including the length and number of sequences from which each was built. (See S2 Text for further information.)

Sample	Enzyme	Read Length	No. Reads	Motif (Table 5)	Logo
E. coli DHB4	F	31	12,058	CCWGG	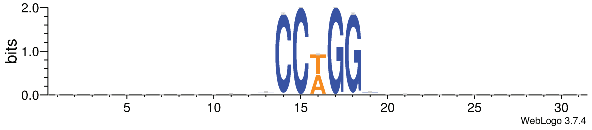
A. calcoaceticus ATCC49823	M	30	1,252	CGCG	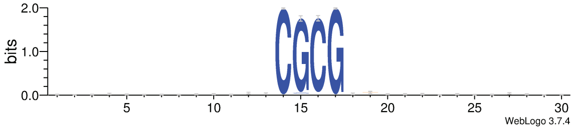
A. calcoaceticus ATCC49823	M	36	755	GATC	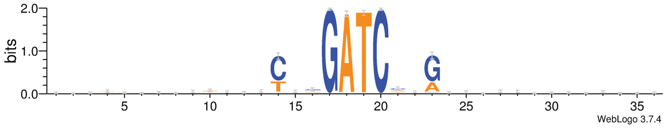
Halorubrum sp. BOL3-1	M	32	3,107	CTCGAG	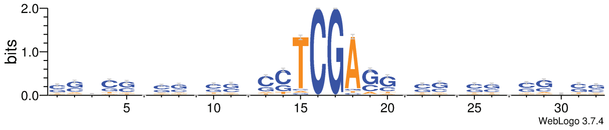
Halorubrum sp. BOL3-1	M	34	650	TGCA	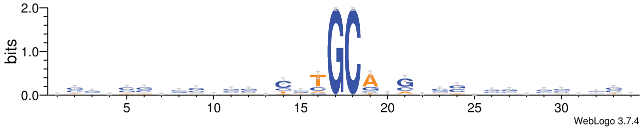
M.HhaI clone	M	31	9,582	CCWGG	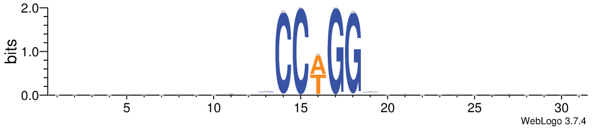
M.HhaI clone	M	32	5,386	GCGC	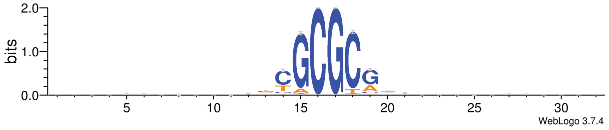
A. variabilis ATCC27893	M	30	1,576	RCCGGY	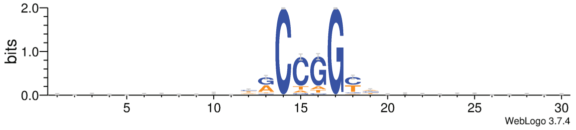
A. variabilis ATCC27893	F	28	495	CGATCG	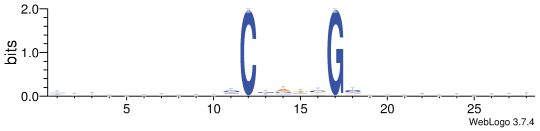
M.AvaII clone	M	35	277	GGWCC	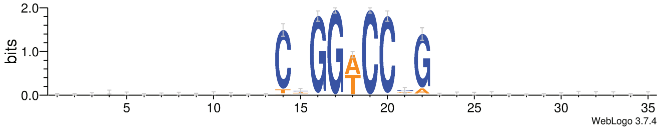

S8 Table. Analysis of the apparent RCHDGY motif in the sequence logo of *A. variabilis* (see S2 Text and S7 Table).

Motif	Sites in Genome	Fraction of Motif Sites	Sites with Reads	Fraction of Sites with Reads
RCHDGY	47373	1.000	1770	0.037
ACAAGC	2238	0.047	32	0.014
GCAAGC	1518	0.032	20	0.013
ACCAGC	2186	0.046	43	0.020
GCCAGC	1233	0.026	19	0.015
ACTAGC	1790	0.038	26	0.015
GCTAGC	1081	0.023	22	0.020
ACAGGC	1175	0.025	13	0.011
GCAGGC	785	0.017	8	0.010
ACCGGC	353	0.007	321	0.909
GCCGGC	361	0.008	309	0.856
ACTGGC	1698	0.036	27	0.016
GCTGGC	1346	0.028	29	0.022
ACATGC	10	0.000	0	0.000
GCATGC	4	0.000	0	0.000
ACCTGC	1518	0.032	27	0.018
GCCTGC	817	0.017	11	0.013
ACTTGC	1728	0.036	18	0.010
GCTTGC	1530	0.032	14	0.009
ACAAGT	1831	0.039	11	0.006
GCAAGT	1647	0.035	15	0.009
ACCAGT	2243	0.047	28	0.012
GCCAGT	1720	0.036	26	0.015
ACTAGT	1440	0.030	15	0.010
GCTAGT	1773	0.037	18	0.010
ACAGGT	1736	0.037	25	0.014
GCAGGT	1531	0.032	23	0.015
ACCGGT	223	0.005	201	0.901
GCCGGT	374	0.008	326	0.872
ACTGGT	2096	0.044	21	0.010
GCTGGT	2236	0.047	31	0.014
ACATGT	12	0.000	0	0.000
GCATGT	8	0.000	0	0.000
ACCTGT	1853	0.039	24	0.013
GCCTGT	1177	0.025	20	0.017
ACTTGT	1805	0.038	15	0.008
GCTTGT	2297	0.048	32	0.014

S9 Table. MFRE cleavage properties of all known m5C motifs.[a]

Motif	Palindrome	Ds Methylated	MspJI	FspEI
AACGTT	+	+		
ACCTGC				
ACGCGT	+	+	1	
ACGT	+	+	0.25	
AGCT	+	+	0.25	
AGGCCT	+	+	0.25	1
CACGTC		+		
CACGTG	+	+	1	
CASTG	+	+		0.0625
CATG	+	+	1	0.0625
CCAG				
CCAGA		+	1	
CCAGA				
CCATGG	+	+		0.0625
CCCGC				
CCCGT				
CCD				
CCGC		+		
CCGC				
CCGCGG	+	+	1	1
CCGG	+	+	0.25	1
CCGG	+	+	1	0.0625
CCNGG	+	+	1	1
CCNGG	+	+	1	0.0625
CCTC				
CCTGA		+	1	
CCTTC				
CCTTC				
CCWGG	+	+	1	1
CCWGG	+	+	1	0.0625
CG	+	+	0.25	0.0625
CGATCG	+	+		0.0625
CGCG	+	+	1	0.0625
CGGCCG	+	+		0.0625
CGGCCG	+	+	0.25	
CGR				
CRCCGGYG	+	+	1	
CTCGAG	+	+	1	0.0625
CTCGAG	+	+	1	
CTCGAR		+	1	
CTGCAG	+	+		0.0625
CTGCAG	+	+	0.25	
CTNAG	+	+	1	0.0625
CYCGRG	+	+	1	0.0625

GACGC		+		
GACGC				
GAGCTC	+	+	0.25	
GAGCTC	+	+	0.25	
GATC	+	+	0.25	
GC	+	+	0.25	
GCAGGC		+	1	
GCCCGGGC	+	+	1	1
GCCGGC	+	+	1	
GCCGGC	+	+		1
GCGATCGC	+	+		
GCGC	+	+	0.25	
GCGCGC	+	+	1	
GCNGC	+	+	0.25	
GCNGC	+	+		
GCNNGC	+	+	1	
GCSGC	+	+		
GCWGC	+	+		
GGATCC	+	+		
GGCC	+	+	0.25	
GGCGCC	+	+		
GGCGGA				
GGGAC		+		
GGGCCC	+	+	0.25	
GGNCC	+	+	0.25	
GGNCC	+	+	0.25	1
GGNNCC	+	+	0.25	
GGTCTC				
GGWCC	+	+	0.25	
GGWCC	+	+	0.25	1
GGYRCC	+	+	0.25	
GKGCMC	+	+	0.25	1 (skew)
GRGCYC	+	+	0.25	
GTCGAC	+	+		
GTCTC				
GTGCAC	+	+	0.25	
GWGCWC	+	+	0.25	
RCATGY	+	+	1	
RCCGGY	+	+	1	
RCCGGY	+	+		1
RCCGGB		+	1	
RGATCY	+	+	0.25	
RGCB		+	0.25	
RGCB				
RGCGCY	+	+		
RGCY	+	+	0.25	
RGGNCCY	+	+	0.25	

RT<u>CG</u>AY	+	+		
TCCG<u>C</u>C				
T<u>CG</u>A	+	+	0.25	
T<u>C</u>TGG				
YA<u>CG</u>TR	+	+	1	
Y<u>C</u>GC<u>G</u>R	+	+	1	
YG<u>C</u>CG<u>G</u>CR	+	+	1	
YG<u>GC</u>CR	+	+	0.25	

[a] Motifs that are palindromic or are methylated on both strands (and therefore addressable by the MFRE technique) are marked with "+". Cleavability by an MFRE is indicated by the fraction of sites cleavable; cleavage recognition elements outside the methylation motif limit the number of cleavable examples to 1/4 for MspJI and 1/16 for FspEI.

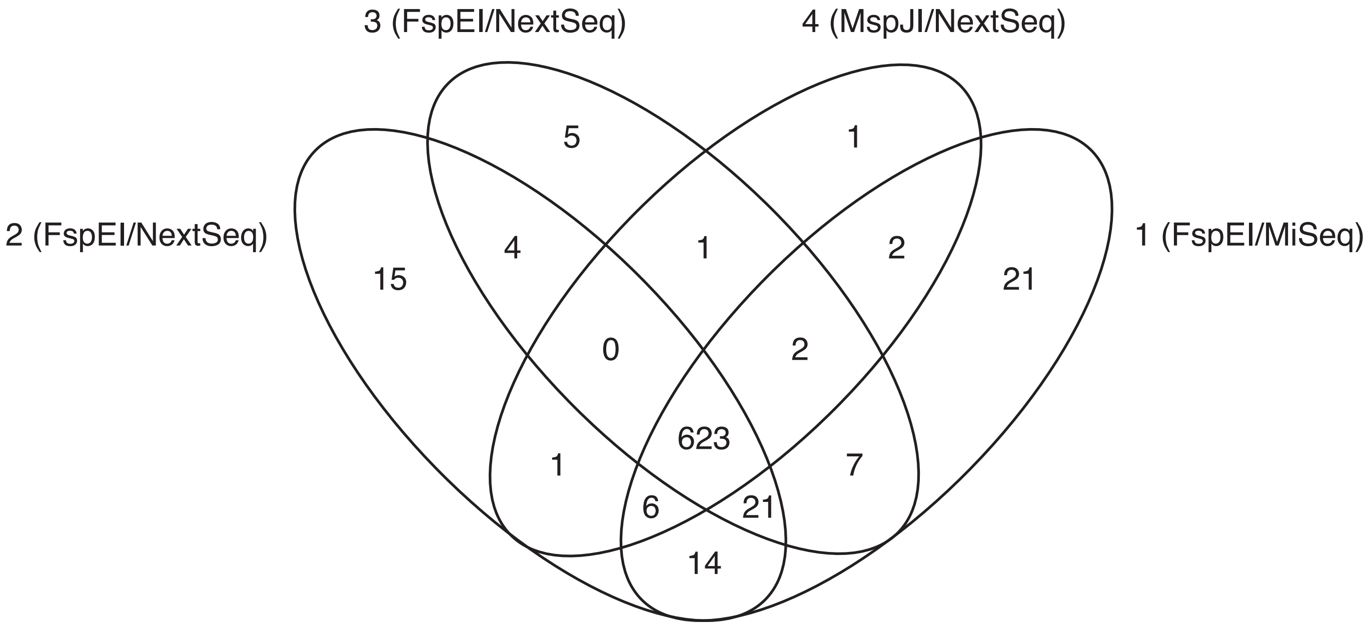

Figure S1. Venn diagram of unrepresented sites from the four libraries of E. coli K-12 DHB4 described in Table 2 (with corresponding numbering). Figures show the number of loci common to one or more libraries.

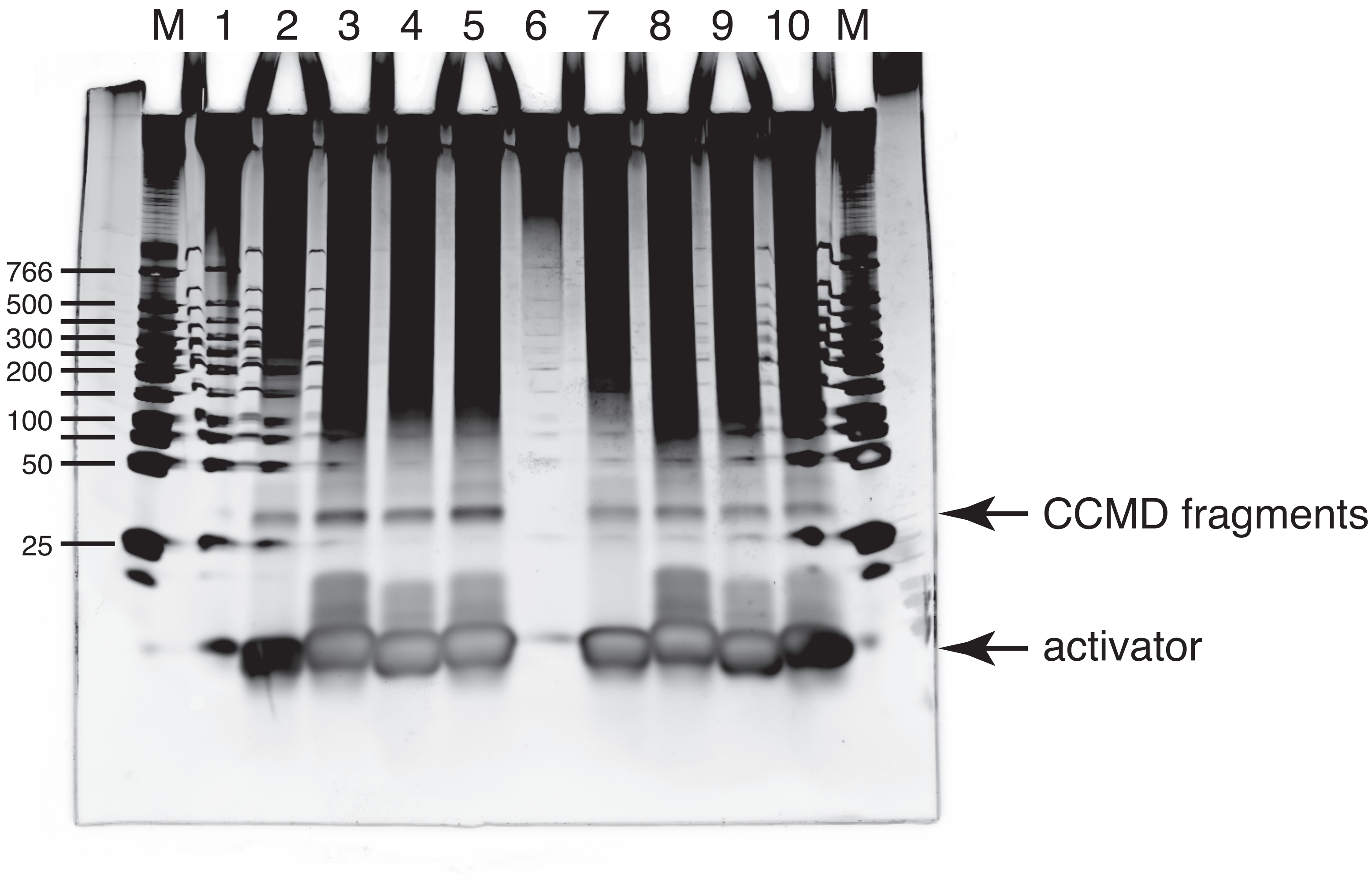

Figure S2. Gel photo (20% TBE-PAGE stained with SYBR Gold) of MFRE digest reactions showing activator and cleavage bands. All reactions were MspJI (1 μL) digests of E. coli DHB4 genomic DNA with 0.525 μM enzyme activator in 40 μL 1x CutSmart buffer, 37˚C 3 hrs. M = Low Molecular Weight DNA Ladder (New England Biolabs); hash marks at left indicate the positions of the 11 bands of the marker, and selected sizes (in bp) are indicated. Lanes 1-5 contained 3 μg genomic DNA, and lanes 6-10 contained 1.5 μg genomic DNA. Activators: none (lanes 1 and 6), standard activator (lanes 2 and 7), activator-N (lanes 3 and 8), activator-U (lanes 4 and 9), or activator-UN (lanes 5 and 10).

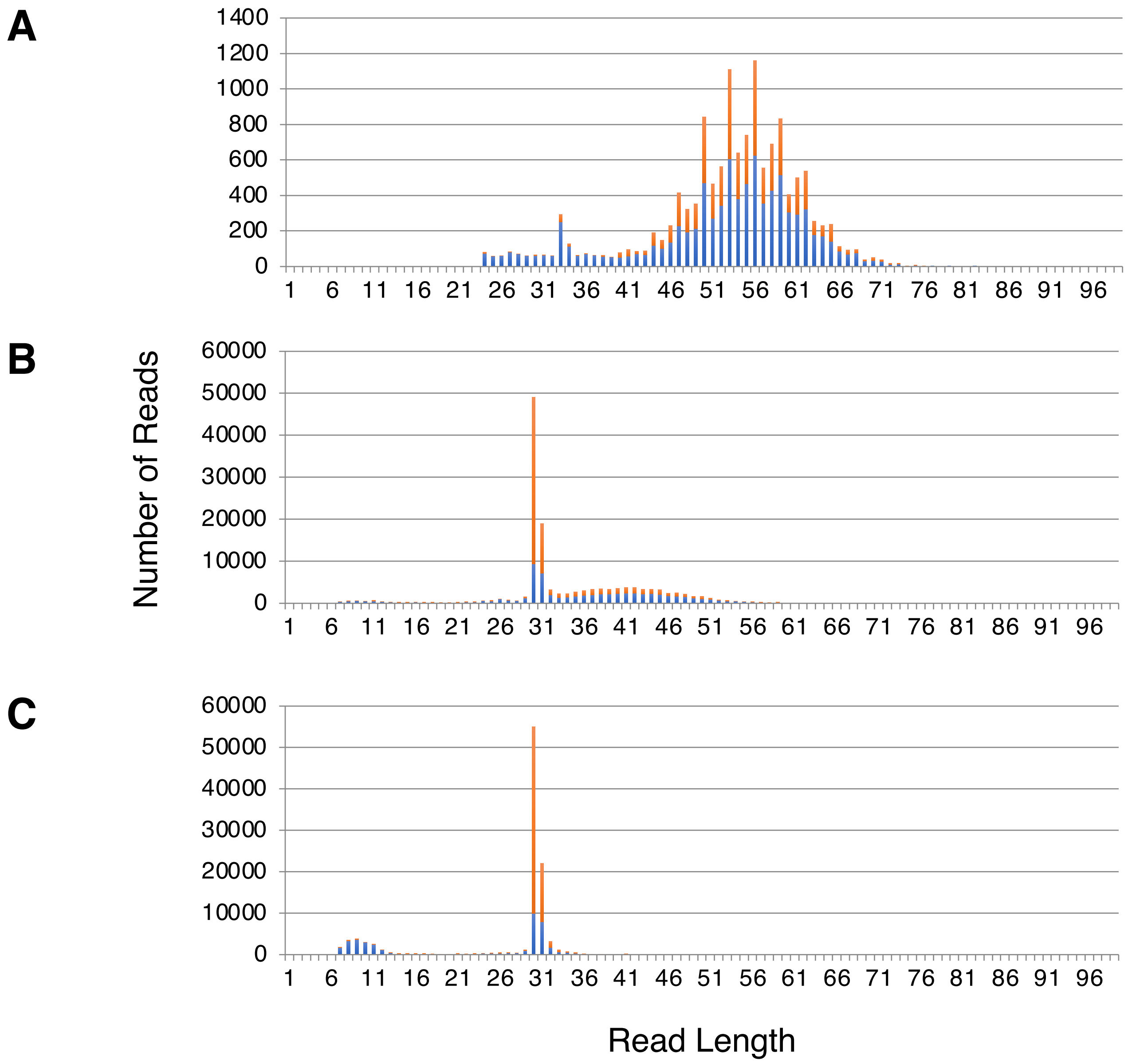

Figure S3. Examples of read distribution with 3 digest cleanup protocols. All 3 samples were digested with MspJI and sequenced on the Ion Torrent platform. For each length, the blue bar indicates the number of unique sequences and the orange bar indicates the number of additional duplicate sequences, so the combined height indicates the number of total reads. **A**. One-step spin-column cleanup, which keeps all fragments, small and large; *Arthrobacter* sp., CCMD length = 34. **B**. Two-step spin-column cleanup, which selects for fragments < 100 bp; *E. coli* DHB4, CCMD length = 31. **C**. Gel-purification of small fragments (20-50 bp range) from 20% polyacrylamide; *E. coli* DHB4, CCMD length = 31.
