# Supplementary material for: Genome-wide identification of 5-methylcytosine sites in bacterial genomes by high-throughput sequencing of MspJI restriction fragments
Source: PLoS One. 2021 May 11;16(5):e0247541. doi: 10.1371/journal.pone.0247541 (PMC8112702; doi:10.1371/journal.pone.0247541)
Supplement: S1 Raw images — (PDF) [file pone.0247541.s002.pdf]

Original gel image for Fig S2.

20% PAGE gel run in 0.5x TBE buffer. Stained 15 min with SYBR Gold (diluted 1:50,000 in the TBE running buffer). Gel imaged on a Typhoon (GE Amersham) with excitation at 488 nm (Cy2 525BP20 filter) and exported as .tif. Excess brightness reduced with ImageJ.

Digest reactions (lanes 2-11 below) were *E. coli* DHB4 genomic DNA digested with MspJI (New England Biolabs) in 40 µL 1x CutSmart buffer plus 0.525 µM enzyme activator, 37˚C 3 hrs. See text for description of the activators.

Sample lanes:

1. Low Molecular Weight DNA Ladder (New England Biolabs #N3233).
2. 3 µg gDNA, no activator.
3. 3 µg gDNA, unmodified activator.
4. 3 µg gDNA, 5'-amino modified activator.
5. 3 µg gDNA, internal T>U modified activator.
6. 3 µg gDNA, both 5'-amino and internal T>U modified activator.
7. 1.5 µg gDNA, no activator.
8. 1.5 µg gDNA, unmodified activator.
9. 1.5 µg gDNA, 5'-amino modified activator.
10. 1.5 µg gDNA, internal T>U modified activator.
11. 1.5 µg gDNA, both 5'-amino and internal T>U modified activator.
12. Low Molecular Weight DNA Ladder (New England Biolabs #N3233).

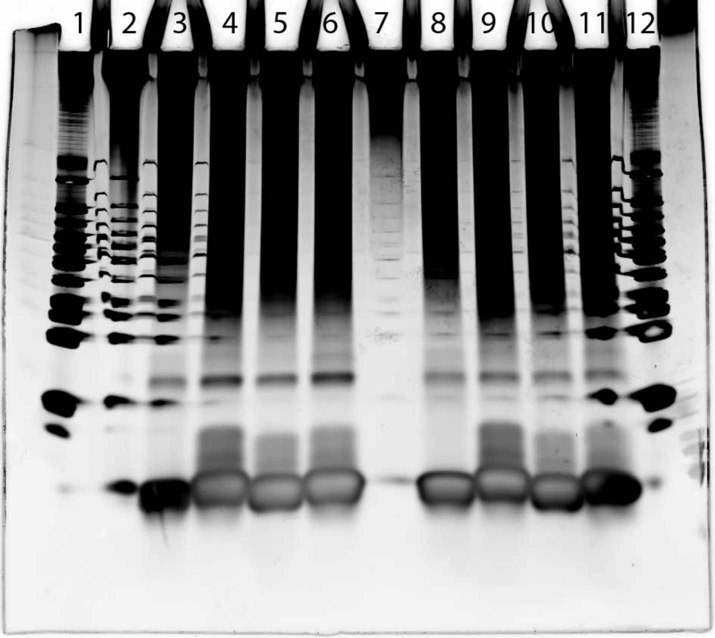

1 2 3 4 5 6 7 8 9 10 11 12
